# Trends in drug resistance codons in *Plasmodium falciparum* dihydrofolate reductase and dihydropteroate synthase genes in Kenyan parasites from 2008 to 2012

**DOI:** 10.1186/1475-2875-13-250

**Published:** 2014-07-02

**Authors:** Dennis W Juma, Angela A Omondi, Luiser Ingasia, Benjamin Opot, Agnes Cheruiyot, Redemptah Yeda, Charles Okudo, Jelagat Cheruiyot, Peninnah Muiruri, Bidii Ngalah, Lorna J Chebon, Fredrick Eyase, Jacob Johnson, Wallace D Bulimo, Hoseah M Akala, Ben Andagalu, Edwin Kamau

**Affiliations:** 1 Global Emerging Infections Surveillance (GEIS) Program, United States Army Medical Research Unit-Kenya (USAMRU-K), Kenya Medical Research Institute (KEMRI) - Walter Reed Project, Kisumu and Nairobi, Kenya

**Keywords:** Malaria, *Plasmodium falciparum* dihydrofolate reductase, *Plasmodium falciparum* dihydropteroate synthase

## Abstract

**Background:**

Sulphadoxine-pyrimethamine (SP), an antifolate, was replaced by artemether-lumefantrine as the first-line malaria drug treatment in Kenya in 2004 due to the wide spread of resistance. However, SP still remains the recommended drug for intermittent preventive treatment in pregnant women and infants (IPT_P/I_) owing to its safety profile. This study assessed the prevalence of mutations in *dihydrofolate reductase* (*Pfdhfr*) and *dihydropteroate synthase* (*Pfdhps*) genes associated with SP resistance in samples collected in Kenya between 2008 and 2012.

**Methods:**

Field isolates collected from Kisumu, Kisii, Kericho and Malindi district hospitals were assessed for genetic polymorphism at various loci within *Pfdhfr* and *Pfdhps* genes by sequencing.

**Results:**

Among the *Pfdhfr* mutations, codons N51**I**, C59**R**, S108**N** showed highest prevalence in all the field sites at 95.5%, 84.1% and 98.6% respectively. *Pfdhfr* S108**N** prevalence was highest in Kisii at 100%. A temporal trend analysis showed steady prevalence of mutations over time except for codon *Pfdhps* 581 which showed an increase in mixed genotypes. Triple *Pfdhfr* N51**I**/C59**R**/S108**N** and double *Pfdhps* A437**G**/ K540**E** had high prevalence rates of 86.6% and 87.9% respectively. The *Pfdhfr*/*Pfdhps* quintuple, N51**I**/C59**R**/S108**N**/A437**G**/K540**E** mutant which has been shown to be the most clinically relevant marker for SP resistance was observed in 75.7% of the samples.

**Conclusion:**

SP resistance is still persistently high in western Kenya, which is likely due to fixation of key mutations in the *Pfdhfr* and *Pfdhps* genes as well as drug pressure from other antifolate drugs being used for the treatment of malaria and other infections. In addition, there is emergence and increasing prevalence of new mutations in Kenyan parasite population. Since SP is used for IPT_P/I_, molecular surveillance and *in vitro* susceptibility assays must be sustained to provide information on the emergence and spread of SP resistance.

## Background

Sulphadoxine-pyrimethamine (SP) has been widely used as first-line treatment for uncomplicated falciparum malaria in many parts of the world including sub-Saharan Africa. It is used alone or in combination with other anti-malarials which has been effective until recently because of high levels of resistance [1,2]. SP has been replaced with other anti-malarials as the first-line treatment in most of sub-Saharan Africa including Kenya [3]. However, SP is still used as intermittent preventive treatment of malaria in pregnancy (IPTp) in sub-Saharan Africa [4]. It was recently recommended for use as intermittent preventive treatment of malaria in infants (IPTi) by the World Health Organization (WHO) in areas with low SP resistance [5]. There has been continued increase in SP resistance albeit being replaced as the first-line treatment [6], which is a major public health concern especially in sub-Saharan Africa [7].

SP resistance is conferred by mutations in *Plasmodium falciparum* dihydrofolate reductase (*Pfdhfr*) and *P. falciparum* dihydropteroate synthase (*Pfdhps*) genes [8,9], whose enzymes are target for pyrimethamine and sulphadoxine respectively. *Pfdhfr* S108**N** mutation has been linked to pyrimethamine resistance. Additional point mutations A16**V**, N51**I** and C59**R** lead to increased resistance [10,11]. High grade pyrimethamine resistance is linked to the occurrence of the I164**L** mutation [12] which has been observed together with the *Pfdhfr* gene triple N51**I**/C59**R**/S108**N** mutant in Southeast Asia and the Americas [12,13]. *Pfdhfr* I164**L** mutation, which is shown to cause rapid spread of antifolate resistance, has been observed in the western parts of Kenya [7,14]. *Pfdhps* A437**G** mutation is mainly associated with sulphadoxine resistance with increased resistance conferred in the presence of additional point mutations S436**A/F/H**, A581**G**, K540**E** and A613**S/T**[9,15]. Differing extents of anti-malarial drug resistance to SP are subject to the varying numbers and combinations of mutations present in the *Pfdhfr* and *Pfdhps* genes [11,16]. Primarily, the *Pfdhfr*/*Pfdhps* N51**I**, C59**R,** S108**N/**A437**G,** K540**E** quintuple mutation has strongly been associated with clinical SP treatment failure [11].

A recent meta-analysis of the frequencies of *Pfdhfr* and *Pfdhps* mutant genotype in African *P. falciparum* parasite populations revealed high prevalence of mutant genotypes along the Kenya-Tanzania border and Malawi [6]. To assess the prevalence of mutations in *Pfdhfr* and *Pfdhps* genes and the impact of introducing SP as the first-line treatment in Kenya, two studies were conducted. A baseline study was conducted in 1999-2000 [17] and the follow-up study in 2003-2005 [18]. The baseline study coincided with the implementation of SP as the first-line treatment whereas the follow-up study coincided with transition to artemether-lumefantrine (AL) as the first-line treatment. The prevalence of isolates containing the quintuple mutant genotype (*Pfdhfr* N51**I**/C59**R**/S108**N**/ *Pfdhps* A437**G**/K540**E**) rose from 21% in the baseline study to 53% in the follow-up study (P < 0.001) [17,18]. The *Pfdhfr* I164**L** mutation was not present in either of the study. Interestingly, a study that analyzed prevalence of the I164**L** mutation in sample isolates collected in the same region in 2002-2008 and 2008-2009 reported a prevalence of 0.6% and 0.8% respectively [19]. There were no mutations at *Pfdhps* codons 581 and 613 at the baseline study but 85% and 61% of the sample isolates in the follow-up study carried mutations at these two codons, respectively [19].

Since SP remains widely used as IPTp and is a possible ACT partner drug as well as IPTi candidate, there is heightened recognition underlying the need to track the prevalence of mutations associated with SP resistance. This study presents the prevalence and trend of mutations in *Pfdhfr* and *Pfdhps* genes in sample isolates collected from 4 different sites in Kenya between 2008 and 2012.

## Methods

### Study sites and sampling collection

This study was conducted after approval by the Kenya Medical Research Institute (KEMRI) and Walter Reed Army Institute of Research (WRAIR) institutional review boards (protocol numbers: KEMRI 1330, WRAIR 1384; Epidemiology of malaria drug sensitivity pattern). The samples used in this study were collected at district hospitals from four regions in Kenya; *viz* Kisumu District Hospital (KDH), Kisii District Hospital (KSI), Kericho District Hospital (KCH) and Malindi District Hospital (MDH) between the years 2008 and 2012. Kisumu is located in the western region of the country at the shores of Lake Victoria. This is a lowland region with holoendemic malaria. Kisii and Kericho are both highland areas also in the western Kenya regions which experiences hypoendemic malaria. Malindi is at the Kenyan coastal lowland region, but has different malaria episodes from Kisumu, which is also at lowland in the western Kenya region [20,21].

Consenting patients aged six months and above, testing positive for uncomplicated *P. falciparum* malaria by rapid diagnostic test (RDT; Parascreen^®^ (Pan/Pf), Zephyr Biomedicals, Verna Goa, India) were allowed to participate in the study. Briefly, 2-3 ml of whole blood was collected from the participants. FTA filter paper (Whatman Inc., Bound Brook, New Jersey, USA) was used to collect 3 blood spots of about 100 μl each for DNA extraction.

### Genotypic analysis for *Pfdhfr* and *Pfdhps*

The DNA was extracted from the FTA filter papers using the QIAamp DNA mini kit; Qiagen protocol (Qiagen, Valencia, CA). Screening for mutations associated with antifolate resistance at codons 16, 50, 51, 59, 108, and 164 of the *Pfdhfr* gene and codons 436, 437, 540, 581, and 613 of the *Pfdhps* gene was performed by nested PCR as previously described [22,23]. After successfully amplifying the target regions, the isolates were purified using Exosap-it^®^ (Affymetrix, Santa Clara, CA) as per the manufacturer’s protocol. Sequencing of the target regions was done on the 3500 xL ABI Genetic analyzer using version 3.1 of the big dye terminator method (Applied Biosystems, Foster City, CA). Contig assembly of the generated sequences was performed using DNA Baser version 3× and the sequences aligned and analysed using BioEdit version 7.1.3.0. All sequences were compared against the *Pfdhfr* (Accession Number; XM_001351443) and *Pfdhps* (Accession Number; XM_001349382) 3D7 reference sequence published at the NCBI database.

### Statistical analysis

Categorical data consisting of genotype polymorphisms within *Pfdhfr, Pfdhps* genes was analyzed as proportions showing rates of frequency. The differences in frequencies of point mutations in the *Pfdhfr, Pfdhps* genes and the combined *Pfdhfr/Pfdhps* genes within and between regions were determined by the Chi-square test. For evaluating trends, the annual frequencies of polymorphisms of individual codons, during the study period were compared. Further, comparison of genotype frequencies of 2003 to 2005 and 2008 to 2012 study periods was performed by Chi-square test. Yates’ correction was applied for the chi-square value, resulting in corrected *P* values. All statistical analyses were performed at the 5% significance level and the corresponding 95% Confidence Interval (CI). Prism 4.0 software (Graph pad Software, San Diego, California, USA) was used for analyses.

## Results

### Prevalence of SNPs in *Pfdhfr* and *Pfdhps* genes

**Figure 1:**
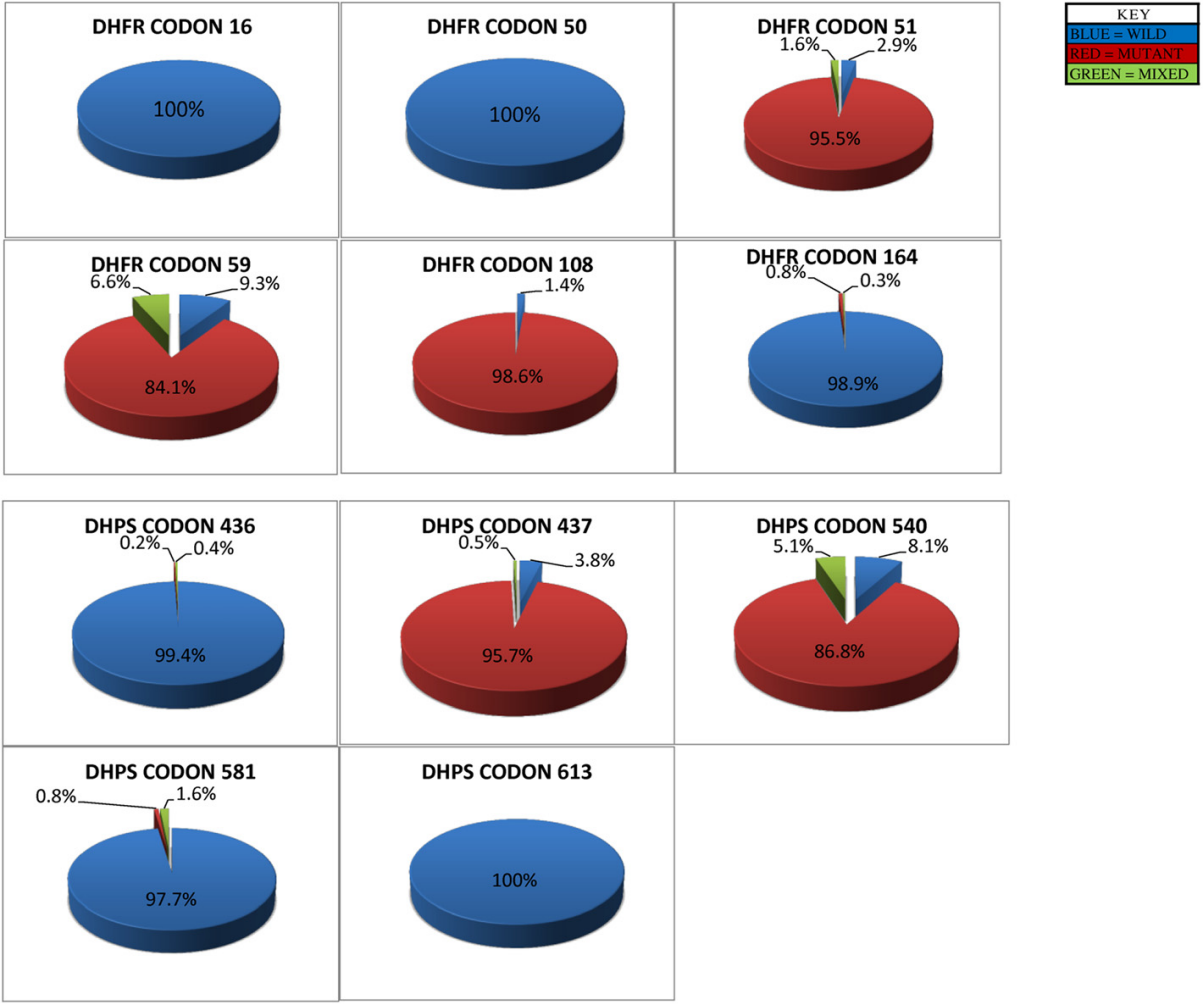
**Prevalence of single nucleotide polymorphism in *Pfdhfr *and *Pfdhps*.** The overall prevalence of genotypes at each codon on the *Pfdhfr* and *Pfdhps* genes were determined over the 5 year period, calculated as a percentage of the total number of samples successfully analyzed over the study period per codon.

A total of 869 archival field isolates collected from KDH, KSI, KCH and MDH between 2008 and 2012 were included in the study of which 822 were successfully sequenced. Eleven SNPs were genotyped, six loci in the *Pfdhfr* gene and five loci in *Pfdhps* gene as shown in the methods section. There were no mutations in *Pfdhfr* gene at codons 16 and 50 as well as *Pfdhps* gene at codon 613. These codons are excluded from any further analysis. Using the criteria that was recently described by Iriemenam *et al*. [19], SNPs were designated as pure (which contains only either wild type or mutant strains) or as mixed (which contained both wild type and mutant alleles based on presence of two major peaks of the chromatograms). Figure 1 shows the overall prevalence of genotypes at each codon. The prevalence of mutation at *Pfdhfr* codons 51, 59 and 108 was 95.5%, 84.1% and 98.6% respectively. There were five (0.8%) sample isolates that carried the pure mutant and 2 (0.3%) samples that had the mixed SNP at *Pfdhfr* codon 164. The prevalence of mutation at *Pfdhps* codons 436, 437, 540 and 581 was 0.2%, 95.7%, 86.8%, and 0.8%.

### Prevalence of mutant alleles in *Pfdhfr* and *Pfdhps* genes per study site

Table 1 shows the prevalence of *Pfdhfr* and *Pfdhps* SNPs per study site for the entire period which isolates were collected. SNPs are classified as wild type, mutant or mixed. The prevalence of mutant allele at *Pfdhfr* codon 108 was highest, with KSI and KDH having 100% and 99.6% of the sample isolates carrying the mutant allele respectively. MDH had the lowest prevalence of mutations in *Pfdhfr* gene at codons 51, 59 and 108 compared to the other sites (P = 0.0052, 0.0002 and 0.0066) respectively. Mutation at *Pfdhfr* codon 164 was present only in samples collected in KDH and KSI. There were five sample isolates which carried the pure mutant (three from KDH and two from KSI), and two sample isolates, one from each site that carried the mixed SNP genotype. In *Pfdhps* gene, the prevalence of mutant allele at *Pfdhps* codon 437 was highest, with KDH and KSI having 98.1% and 97.9% of the sample isolates carrying the mutant allele respectively. Mutant and mixed SNPs at *Pfdhps* codon 581 was present in sample isolates collected in KDH, KSI and KCH. MDH site had the lowest prevalence of mutant alleles at codons 437 and 540 compared to the other sites.

**Table 1:** **Prevalence of single nucleotide polymorphism in *Pfdhfr *** **and *Pfdhps *** **per site**

		**DHFR prevalence per site**						**DHPS prevalence per site**				
	**CODON**	**16**	**50**	**51**	**59**	**108**	**164**	**S436A/F/H**	**437**	**540**	**581**	**613**
		% (n)	% (n)	% (n)	% (n)	% (n)	% (n)	% (n)	% (n)	% (n)	% (n)	% (n)
KDH	WILD	100 (266)	100 (289)	1.4 (4)	3.5 (10)	0.3 (1)	98.7 (302)	100 (256)	1.5 (4)	7.5 (21)	97.2 (273)	100 (284)
	MUTANT	0 (0)	0 (0)	96.9 (279)	92.7 (267)	99.6 (296)	1 (3)	0 (0)	98.1 (264)	91.1 (256)	0.4 (1)	0 (0)
	MIXED	0 (0)	0 (0)	1.7 (5)	3.8 (11)	0 (0)	0.3 (1)	0.4 (1)	0.4 (1)	1.4 (4)	2.5 (7)	0 (0)
KSI	WILD	100 (152)	100 (155)	1.3 (2)	11.0 (17)	0 (0)	98.1 (155)	100 (87)	1.1 (1)	9.9 (14)	96.7 (146)	100 (117)
	MUTANT	0 (0)	0 (0)	96.9 (152)	82.5 (127)	100 (156)	1.32 (2)	0 (0)	97.9 (91)	89.4 (126)	2.0 (3)	0 (0)
	MIXED	0 (0)	0 (0)	1.9 (3)	6.5 (10)	0 (0)	0.6 (1)	0 (0)	1.1 (1)	0.7 (1)	1.3 (2)	0 (0)
KCH	WILD	100 (93)	100 (93)	2.2 (2)	7.7 (6)	2.1 (2)	100 (93)	98.9 (89)	2.2 (2)	3.8 (4)	98.1 (102)	100 (104)
	MUTANT	0 (0)	0 (0)	97.9 (91)	73.1 (57)	97.9 (92)	0 (0)	0 (0)	96.7 (87)	90.4 (94)	1.0 (1)	0 (0)
	MIXED	0 (0)	0 (0)	0 (0)	19.2 (15)	0 (0)	0 (0)	1.1 (1)	1.1 (1)	5.8 (6)	1.0 (1)	0 (0)
MDH	WILD	100 (99)	100 (103)	10.7 (11)	24.3 (25)	5.8 (6)	100 (103)	98.9 (98)	13.2 (14)	11.9 (12)	100 (104)	100 (107)
	MUTANT	0 (0)	0 (0)	87.4 (90)	70.9 (73)	94.2 (97)	0 (0)	1.0 (1)	86.8 (92)	86.1 (87)	0 (0)	0 (0)
	MIXED	0 (0)	0 (0)	1.9 (2)	4.9 (5)	0 (0)	0 (0)	0 (0)	0 (0)	2.0 (2)	0 (0)	0 (0)

Prevalence of *Pfdhfr* and *Pfdhps* wildtype, mutant and mixed genotypes per study site for the entire period was calculated as a percentage of the total number of samples successfully analysed over the study period per codon per the specific site.

### Temporal trends of SNPs in *Pfdhfr* and *Pfdhps* genes

Additional file 1 shows the temporal trends of mutations in *Pfdhfr* and *Pfdhps* genes of samples collected from all the four sites. Samples collected from KDH and KCH displayed steady prevalence over time at all codons except for codon 581 in KDH which the prevalence of mixed genotype increased from 1.2% in 2009 to 15.6% in 2012. However, MDH and to lesser extent KCH showed changing temporal trends. At MDH, samples collected in 2011 had lower prevalence of mutation in *Pfdhfr* codons 51, 108 and *Pfdhps* codon 437 compared to samples collected in 2010 and 2012. Chi-square analysis showed that the prevalence of *Pfdhfr* codon 108 and *Pfdhps* codon 437 mutant genotypes were significantly different (*P* = 0.016 and 0.039) respectively. Although it did not reach statistical significance, there was a downward trend of the prevalence of mutation at codon 59 and 540 (*P* = 0.875 and 0.5146), respectively, for samples collected in KCH and MDH.

### Prevalence of *Pfdhfr* and *Pfdhps* genotypes

Prevalence of *Pfdhfr* and *Pfdhps* genotypes was based on criteria that were originally described by Kublin *et al.*[11] and recently by Iriemenam *et al.*[19]. Genotype analysis was done only on those samples that were successfully analysed at all loci. Mixed genotypes containing two alleles at a single locus were pooled with pure mutant (single) alleles for analysis. *Pfdhfr* genotypes were based on mutations at codons 51, 59, and 108 which were classified as wild type, single, double, and triple. *Pfdhps* genotypes were based on mutations at codons 437 and 540 which were classified as wild type, single, and double. Combined *Pfdhfr* (51, 59, and 108) and *Pfdhps* (437, 540) genotypes were defined as wildtype, single, double, triple, quadruple and quintuple. Quintuple genotype represented infections in which all the five mutations were present. Additional combinations which contained mutation at *Pfdhfr* codon 164, *Pfdhps* codons 436 and 581 were genotyped as well.

Additional file 2 shows the prevalence of genotypes in *Pfdhfr* or *Pfdhps* genes. In *Pfdhfr* gene, triple mutant (N51**I**/C59**R**/S108**N**) was the most prevalent at 86.6% in all sites combined whereas single mutant (C59**R**), double mutant (N51**I**/C59**R**) and the quadruple mutant (N51**I**/C59**R**/S108**N**/I164**L)** were the least prevalent at 0.26% each. When broken further per site, the triple mutant was most prevalent in KDH at 93.7% whereas least prevalent in MDH at 67.9%. There was no genotype observed which contained *Pfdhfr* N51**I** single mutant. In the *Pfdhps* gene, double mutant (A437**G**/K540**E**) was the most prevalent at 87.9% whereas single mutant (S436**A**) was the least prevalent at 0.26% (one sample). Interestingly, two of the four samples in triple mutant 436**/**437/540 carried S436**F** and S436**H** amino acid mutation and not the S436**A** which has been shown to be more prevalent mutation [19] . These mutations occurred either as a triple S436**F**/A437**G**/K540**E** or S436**H**/A437**G**/K540**E** mutations. Of note is prevalence of A581**G** mutant which has been rare in the region, but has been seen to emerge in recent years [19]. The A581**G** mutant appeared as a genotype in combination with the A437**G** mutation in 0.78% of the isolates analysed. It was also observed as a triple A437**G**/K540**E**/A581**G** mutant genotype in 1.6% of the samples. Interestingly, A581**G** mutant was not observed as a single mutant, but appeared in the presence of other mutations on the gene.

Additional file 3 shows the prevalence of genotypes in combined *Pfdhfr* and *Pfdhps* genes. In all the sites, the quintuple mutant (*Pfdhfr*/*Pfdhps* N51**I**,C59**R,**S108**N/**A437**G,**K540**E**) was the most prevalent at 75.71% whereas the least prevalent was quadruple mutant (*Pfdhfr*/*Pfdhps* N51**I**,C59**R**,S108**N/**S436**A**) and sextuple mutant (*Pfdhfr*/*Pfdhps* N51**I**,C59**R**,S108**N**, I164**L/**A437**G,**K540**E**) at 0.26%. When broken down per site, KCH had the highest prevalence of the quintuple mutant at 89.6% whereas MDH was the lowest at 54.3%.

### Temporal trends of genotypes in *Pfdhfr* and *Pfdhps* genes

Additional file 4 shows percent temporal prevalence of genotypes from 2008-2012. In *Pfdhfr* gene, the wildtype of the genotype analysed is N51,C59,S108,I164 (NCSI) and in the *Pfdhps* gene, the wildtype of the genotype analysed is S436,A437,K540,A581 (SAKA). For *Pfdhfr* gene, the prevalence of wildtype genotype increased slightly from 2008 to 2011, but was not present in 2012. The ***IRN*** I genotype (triple mutant, 51I,59R,108 N) was the most prevalent but decreased from 92.5% in 2008 to 72.3% in 2012. For *Pfdhps* gene, the prevalence of the wildtype genotype was not steady overtime. The S ***GE*** A genotype (double mutant, 437G, 540E) was the most prevalent. However, unlike the *Pfdhfr* ***IRN*** I genotype which declined over time, the *Pfdhps* S ***GE*** A genotype remained relatively stable over time.

## Discussion

The *Pfdhfr*/*Pfdhps* N51**I**, C59**R,** S108**N/**A437**G,** K540**E** quintuple mutant genotype is strongly associated with clinical SP treatment failure [11]. Using sample isolates collected from general patient population, quintuple mutant genotype was shown to be the most prevalent combined genotype at 75.7%. When further broken down per site, KCH and KDH had the highest prevalence at 89.6% and 80.6%, respectively whereas MDH had the lowest prevalence at 54.3% (see Additional file 3). In other studies conducted in Kenya using sample isolates collected in Kisumu, Iriemenam *et al*. [19] and Spalding *et al.*[18] showed prevalence of the quintuple mutant genotype to be at 88% and 53% respectively. The difference in prevalence can be explained by the period and patient population which sample isolates were collected from. Iriemenam *et al.* study used sample isolates collected in 2008-2009 from pregnant patient population whereas Spalding *et al.* used general patient population sample isolates collected in 2003-2005. In the current study, the prevalence of the quintuple mutant phenotype is similar to that from Iriemenam *et al.* study most likely because the samples were collected around the same period. The small difference seen can be attributed to the difference in patient population that sample isolates were collected from and is also reflective of the malaria attack rates found in those locations. The attack rate has been significantly lower at the coast over the last five years, due mostly to the use of bed nets and other environmental controls [21].

Although 2003-2005 marked the period which SP was discontinued and AL introduced as first-line treatment for malaria, the prevalence of *Pfdhfr*/*Pfdhps* N51**I**, C59**R,** S108**N/**A437**G,** K540**E** quintuple mutant genotype which is strongly associated with clinical SP treatment failure continued to increase at an alarming rate during this period. Iriemenam *et al.* and Spalding *et al.* studies observed the prevalence of the quintuple mutant genotype increase from 7% and 21% in sample isolates collected in 1996-2000 and 1999-2000 to 88% and 53% in samples collected in 2008-2009 and 2003-2005 respectively. In the current study, the prevalence of quintuple mutant genotype in KDH (western Kenya) was 80.6%. These studies indicate that there is a fixation of the sulphadoxine-pyrimethamine resistant alleles in parasite population from western Kenya. Such fixation has also been observed in Southeast Asia [23], contrary to what has been reported in the Amazon region of Peru where a decline in sulphadoxine-pyrimethamine-resistant alleles was reported five years after change in drug policy [24]. Fixation of the resistance alleles have been attributed to parasites that may have acquired additional compensatory mutations [25]. The fitness cost imposed by the mutations does not impact the parasite and the parasite populations are fixed for resistance with no competing sensitive parasite. There is also the probable presence of additional selective pressure from antifolate drugs such as cotrimoxazole (trimethoprim-sulphamethoxazole) [26,27]. In addition to widespread use of cotrimoxazole, SP continues to be widely available at the local pharmacies (chemists) with self-report SP use still high regardless of AL being the recommended first-line malaria treatment in Kenya. Experimental or epidemiological evidence however will be required to show an association between cotrimoxazole and the occurrence of *Pfdhps/Pfdhfr* mutations in *P. falciparum.* In a recently concluded randomized trial, one group of HIV-infected individuals discontinued cotrimoxazole prophylaxis for 12 months whereas the second group continued with cotrimoxazole prophylaxis (unpublished data, personal communication with Dr. Christina Polyak). Comparison of the prevalence of malaria and mutations in *Pfdhps/Pfdhfr* genes in the group that discontinued cotrimoxazole prophylaxis to the group that continued will provide evidence of any association between cotrimoxazole and the occurrence of *Pfdhps/Pfdhfr* mutations in *P. falciparum.* Other factors such as host immunity and transmission intensity might attribute to the fixation of *Pfdhps/Pfdhfr* mutant alleles in parasites in western Kenya [19].

Mutations at *Pfdhps* codon 581 and *Pfdhfr* codon 164 have been linked to high rate of therapeutic failures in Southeast Asia, South America [23,28] and more recently in Africa [29-31]. Recent studies have shown emergence and increase of the triple mutant allele (A437G/K540E/A581G) in Tanzania [29,32]. Alifrangis *et al.*[32] showed that the prevalence of mutation at *Pfdhps* codon 581 increased significantly from 12% in 2003 to 56% in 2007, resulting in an increase in the triple mutant *Pfdhps* haplotype (A437G/K540E/A581G) from 8% to 32%. In studies conducted in Kenya, Iriemenam *et al.*[19] and Spalding *et al.*[18] found the prevalence of triple mutant *Pfdhps* haplotype to be 5.3% and 15% respectively whereas in this study, the prevalence was 1.6% (see Additional file 2). Sample isolates from Iriemenam *et al.* study and the current study were collected around the same period (2008-2009 vs. 2008-2012) and showed similar prevalence of the triple mutant *Pfdhps* haplotype. However, sample isolates from Spalding *et al.* study collected in 2003-2005 showed significantly high prevalence of mutations at *Pfdhps* codon 581 (85%), only three years after mutations at the *Pfdhps* codon did not exist [17]. This is unexpected since Iriemenam *et al.* study and the current study, which used sample isolates collected at later time period showed lower prevalence of the triple mutant *Pfdhps* haplotype (A437G/K540E/A581G), yet prevalence of this mutation has been on a steady increase during the SP treatment period [19]. However the prevalence of the triple mutant *Pfdhps* haplotype from Spalding *et al.* study is similar to that seen in Tanzania [32]. Mutation at *Pfdhfr* codon 164 remains low. The study done using samples collected from western Kenya between 1992 and1999 by McCollum *et al.*[33] indicated very low prevalence of *Pfdhfr* wild type alleles while majority had double *Pfdhf*r 51/108, 59/108 and *Pfdhps* 437/540 mutations (50%, 27% and 57% respectively). *Pfdhps* wild type alleles were relatively high at 34% at that period. This was a period before SP was the first line antimalarial treatment and indicated initial development of resistant traits in the population.

This is the first study which compares prevalence of drug resistance mutant genotypes in *Pfdhfr* and *Pfdhps* genes in sample isolates collected from western and coastal regions of Kenya. Sample isolates collected from Malindi (MDH) showed significant difference in prevalence of drug resistance alleles in *Pfdhfr* and *Pfdhps* genes compared to sample isolates collected from western Kenya (Table 2). Interestingly, mutation in *Pfdhfr* codon 51 and 108 as well as *Pfdhps* codon 437 decreased in 2011 compared to 2010, but increased in 2012. Mutation in *Pfdhps* codon 540 remained the same in 2010 and 2011, but increased significantly in 2012 (*P* = 0.0196). To the contrarily, *Pfdhfr* codon 59 significantly decreased from 2010 to 2012 (*P* = 0.0064). The prevalence and the temporal trends of mutations in *Pfdhfr* and *Pfdhps* genes in Malindi represent interesting dynamics which could be driven by a number of factors including natural phenomenon which is yet to be determined. The prevalence of malaria is generally on a steady decline in the Kenyan coast (personal communication with Dr. Kevin Marsh) [34] which might result to changes in the immunity of the population in this region. Also, the dip in prevalence seen in 2011 might be due to environmental conditions such as weather patterns which can impact transmission intensity hence the prevalence of drug resistance genotype. Interesting however, mutation at *Pfdhfr* codon 59 showed a steady decline from 2010 to 2012. Further studies are required to further elucidate the dynamics of mutations in *Pfdhfr* and *Pfdhps* genes in Kenyan coast.

**Table 2:** P values indicating significance in statistical differences, P = 0.05

		**Western Kenya (n = 306)**	**Western Kenya (n = 306) vs MDH (n = 81)**
Pfdhfr codons	16	1.0000	1.0000
	50	1.0000	1.0000
	51	0.7762	0.0007
	59	0.0904	<0.0001
	108	0.3642	0.0240
	164	0.3629	0.2571
Pfdhps codons	436	0.3629	0.2964
	437	0.4068	<0.0001
	540	0.2323	0.2259
	581	0.7092	0.2638
	613	1.0000	1.0000

*P* value of the mutant prevalence of each codon was calculated. Comparison was done in samples from western Kenya study sites (KDH, KSI, and KCH) and also between the western Kenya sites verses the coastal study site (MDH) to determine statistical differences in prevalence between the two geographical regions.

## Conclusion

The data presented in this study confirms the fixation of key mutations in the *Pfdhfr* and *Pfdhps* genes conferring resistance to SP. It also shows that there is emergence and increasing prevalence of new mutations in the Kenyan parasite population. The adoption of SP in IPTp might only be playing a minor role in these observations. The main contributors of the antifolate drug pressure are likely to be over the counter SP self-medication and the use of cotrimoxazole in management of HIV patients. Apart from discouraging SP use for symptomatic malaria treatment, further evaluation should be undertaken to show effect of the highly prevalent *Pfdhfr/Pfdhps* quintuple mutant combining with the emerging *Pfdhfr* 164 and/or the *Pfdhps* 581 on the efficacy of IPTp or IPTi. A continuous molecular surveillance accompanied by *in vitro* susceptibility testing will provide valuable information urgently needed to control or reduce spread of SP resistance.

## Competing interest

The authors declare that they have no competing interests.

## Authors’ contributions

DWJ was involved in laboratory oversight, manuscript writing, data analysis and interpretation. AOA carried out molecular assays, drafting the manuscript or revising it critically for important intellectual content. LI and BO participated in data analysis, manuscript revising & molecular assays. ARC was involved in data analysis, revising it critically for important intellectual content. RY participated in data analysis & molecular assays. CO participated in molecular assays, acquisition of data. JC, PM, BN and LC were involved in molecular assays, and data interpretation. FLE was involved in laboratory oversight, revising manuscript critically for important intellectual content. WDB - Manuscript writing, conception and design, data analysis and interpretation. HMA - Data analysis & Manuscript development. JDJ, BA and EK were involved in protocol oversight, Manuscript writing, conception and design, data analysis and interpretation. All authors have read and approved the final version of the manuscript.

## Supplementary Material

Additional file 1 **Prevalence of single nucleotide polymorphism in Pfdhfr and Pfdhps Per year (n = Number of isolates having that genotype).** Description: The data shows the frequency of the individual codon genotypes over the study period per site. Click here for file

Additional file 2 **Prevalence of combined mutation in the Pfdhfr and Pfdhps genes.** Description: The data presented represent prevalence of haplotypes in the *Pfdhfr* and *Pfdhps* genes. Click here for file

Additional file 3 Prevalence of Pfdhfr/Pfdhps combined mutations per site (SITE MUTATION/SITE TOTAL*100). Description: The data represents the prevalence of mutation occurring in both *Pfdhfr* and *Pfdhps* genes simultaneously. Click here for file

Additional file 4 **Percentage temporal prevalence of genotypes.** Description: The data shows average genotype prevalence of all the study sites over the period under investigation. The genotypes analysed for *Pfdhfr* were N51I, C59R, S108N and I164L while for *Pfdhps* were S436A, A437G, K540E and A581G in that order. Amino acid codes before the loci position indicate the wildtype while the ones indicated after the loci position are the mutations. In the table, the genotypes without neither bold nor underlined amino acid codes are the wild type while the underlined and bold are the mutant alleles. Click here for file
