# Supplementary material for: Trends in drug resistance codons in Plasmodium falciparum dihydrofolate reductase and dihydropteroate synthase genes in Kenyan parasites from 2008 to 2012
Source: Malar J. 2014 Jul 2;13:250. doi: 10.1186/1475-2875-13-250 (PMC4094641; doi:10.1186/1475-2875-13-250)
Supplement: Additional file 1 — Prevalence of single nucleotide polymorphism in Pfdhfr and Pfdhps Per year (n = Number of isolates having that genotype). Description: The data shows the frequency of the individual codon genotypes over the study period per site. [file 1475-2875-13-250-S1.pdf]

Additional file 1: Prevalence of single nucleotide polymorphism in Pfdhfr and Pfdhps Per year (n = Number of isolates having that genotype)

DHFR PREVALENCE PER YEAR

		16					50					51				
		2008 % (n)	2009 % (n)	2010 % (n)	2011 % (n)	2012 % (n)	2008 % (n)	2009 % (n)	2010 % (n)	2011 % (n)	2012 % (n)	2008 % (n)	2009 % (n)	2010 % (n)	2011 % (n)	2012 % (n)
**KDH**	**WILD**	100 (47)	100 (62)	100 (46)	100 (55)	100 (56)	100 (49)	100 (66)	100 (54)	100 (60)	100 (60)	4.2 (2)	1.5 (1)	1.9 (1)	0	0
	**MUTANT**	0	0	0	0	0	0	0	0	0	0	91.7 (44)	97 (64)	94.4 (51)	100 (60)	100 (60)
	**MIXED**	0	0	0	0	0	0	0	0	0	0	4.2 (2)	1.5 (1)	3.7 (2)	0	0
**KSI**	**WILD**	100 (26)	100 (36)	100 (29)	100 (33)	100 (28)	100 (28)	100 (36)	100 (30)	100 (33)	100 (28)	3.6 (1)	0	3.3 (1)	2.9 (1)	0
	**MUTANT**	0	0	0	0	0	0	0	0	0	0	96.4 27)	100 (36)	93.3 (28)	94.3 (33)	100 (28)
	**MIXED**	0	0	0	0	0	0	0	0	0	0	0	0	3.3 (1)	2.9 (1)	0
**KCH**	**WILD**	100 (19)	100 (21)	100 (20)	100 (15)	100 (18)	100 (19)	100 (21)	100 (20)	100 (15)	100 (18)	0	0	10 (2)	0	0
	**MUTANT**	0	0	0	0	0	0	0	0	0	0	100 (19)	100 (21)	90 918)	100 (15)	100 (18)
	**MIXED**	0	0	0	0	0	0	0	0	0	0	0	0	0	0	0
**MDH**	**WILD**			100 (25)	100 (22)	100 (52)			100 (29)	100 (22)	100 (52)			6.9 (2)	18.2 (4)	9.6 (5)
	**MUTANT**			0	0	0			0	0	0			89.7 (26)	77.3 (17)	90.4 (47)
	**MIXED**			0	0	0			0	0	0			3.4 (1)	4.5 (1)	0

		59					108					164				
		2008 % (n)	2009 % (n)	2010 % (n)	2011 % (n)	2012 % (n)	2008 % (n)	2009 % (n)	2010 % (n)	2011 % (n)	2012 % (n)	2008 % (n)	2009 % (n)	2010 % (n)	2011 % (n)	2012 % (n)
**KDH**	**WILD**	2 (1)	7.7 (5)	0	3.3 (2)	3.3 (25)	0	1.5 (1)	0	0	0	98.1 (52)	100 (69)	100 (60)	100 (64)	95 (57)
	**MUTANT**	85.7 (42)	89.2 (58)	98.1 (53)	95 (57)	95 (57)	100 (49)	98.5 (67)	100 (56)	100 (62)	100 (62)	1.9 (1)	0	0	0	3.3 (2)
	**MIXED**	12.2 (6)	3.1 (2)	1.9 (1)	1.7 (1)	1.7 (1)	0	0	0	0	0	0	0	0	0	1.7 (1)
**KSI**	**WILD**	7.1 (2)	5.7 (2)	10 (3)	18.2 (6)	14.3 (4)	0	0	0	0	0	96.6 (28)	97.2 (35)	96.7 (29)	100 (33)	100 (30)
	**MUTANT**	85.7 (24)	85.7 (30)	86.7 (26)	72.7 (24)	82.1 (23)	100 (28)	100 (36)	100 (30)	100 (33)	100 (29)	0	2.8 (1)	3.3 (1)	0	0
	**MIXED**	7.1 (2)	8.6 (3)	3.3 (1)	9.1 (3)	3.6 (1)	0	0	0	0	0	3.4	0	0	0	0
**KCH**	**WILD**	0	25 (2)	5.6 (1)	6.7 (1)	11.1 (2)	0	0	10 (2)	0	0	100 (19)	100 (21)	100 (20)	100 (15)	100 (18)
	**MUTANT**	63.2 (12)	37.5 (3)	88.9 (16)	86.7 (13)	72.2 (13)	100 (19)	100 (21)	90 (18)	100 (16)	100 (18)	0	0	0	0	0
	**MIXED**	36.8 (7)	37.5 (3)	5.6 (1)	6.7 (1)	16.7 (3)						0	0	0	0	0
**MDH**	**WILD**			3.4 (1)	27.3 (6)	34.6 (18)			0	18.2 (4)	3.8 (2)			100 (29)	100 (22)	100 (52)
	**MUTANT**			93.1 (27)	68.2 (15)	59.6 (31)			100 (29)	81.8 (18)	96.2 (50)			0	0	0
	**MIXED**			3.4 (1)	4.5 (1)	5.8 (3)			0	0	0			0	0	0

DHPS PREVALENCE PER YEAR

		436					437					540				
		2008 % (n)	2009 % (n)	2010 % (n)	2011 % (n)	2012 % (n)	2008 % (n)	2009 % (n)	2010 % (n)	2011 % (n)	2012 % (n)	2008 % (n)	2009 % (n)	2010 % (n)	2011 % (n)	2012 % (n)
**KDH**	**WILD**	98.8 (80)	100 (76)	100 (54)	100 (24)	100 (22)	1.2 (1)	2.5 (2)	1.8 (1)	0	0	8.5 (7)	7.5 (6)	7.3 (4)	11.8 (4)	0
	**MUTANT**	0	0	0	0	0	98.8 (81)	96.3 (78)	98.2 (54)	100 (28)	100 (23)	90.2 (74)	88.8 (71)	92.7 (51)	88.2 (30)	100 (30)
	**MIXED**	1.2 (1)	0	0	0	0	0	1.2 (1)	0	0	0	1.2 (1)	3.8 (3)	0	0	0
**KSI**	**WILD**	100 (14)	100 (15)	100 (19)	100 (19)	100 (20)	0	0	4.5 (1)	0	0	12.5 (3)	12.5 (4)	17.2 (5)	3.7 (1)	3.4 (1)
	**MUTANT**	0	0	0	0	0	100 (14)	100 (16)	91 (20)	100 (20)	100 (21)	87.5 (21)	84.4 (27)	82.8 (24)	96.3 (26)	96.6 (28)
	**MIXED**	0	0	0	0	0	0	0	4.5 (1)	0	0	0	3.1 (1)	0	0	0
**KCH**	**WILD**	93.3 (14)	100 (18)	100 (19)	100 (18)	100 (20)	0	0	10.5 (2)	0	0	4.5 (1)	0	8.7 (2)	5.9 (1)	0
	**MUTANT**	0	0	0	0	0	100 (14)	100 (18)	89.5 (17)	100 (19)	100 (19)	86.4 (19)	95 (19)	87 (20)	88.2 (15)	95.5 (21)
	**MIXED**	6.7 (1)	0	0	0	0	0	0	0	0	0	9.1 (2)	5 (1)	4.3 (1)	5.9 (1)	4.5 (1)
**MDH**	**WILD**			100 (26)	95 (19)	100 (53)			20.7 (6)	23.8 (5)	5.4 (3)			21.4 (6)	22.7 (5)	2 (1)
	**MUTANT**			0	5	0			79.3 (23)	76.2 (16)	94.6 (53)			75 (21)	77.3 (17)	96 (49)
	**MIXED**			0	0	0			0	0	0			3.6 (1)	0	2 (1)

		581					613				
		2008 % (n)	2009 % (n)	2010 % (n)	2011 % (n)	2012 % (n)	2008 % (n)	2009 % (n)	2010 % (n)	2011 % (n)	2012 % (n)
**KDH**	**WILD**	98.8 (80)	98.8 (80)	98.2 (56)	100 (30)	84.4 (27)	100 (81)	100 (82)	100 (58)	100 (32)	100 (31)
	**MUTANT**	0	0	1.8 (1)	0	0	0	0	0	0	0
	**MIXED**	1.2 (1)	1.2 (1)	0	0	15.6 (5)	0	0	0	0	0
**KSI**	**WILD**	100 (24)	94.6 (35)	97.2 (35)	96.2 (25)	96.4 (27)	100 (16)	100 (28)	100 (31)	100 (20)	100 (22)
	**MUTANT**	0	0	2.8 (1)	3.8 (1)	3.6 (1)	0	0	0	0	0
	**MIXED**	0	5.4 (2)	0	0	0	0	0	0	0	0
**KCH**	**WILD**	100 (22)	100 (22)	100 (21)	94.7 (18)	95 (19)	100 (21)	100 (20)	100 (23)	100 (19)	100 (21)
	**MUTANT**	0	0	0	5.3 (1)	0	0	0	0	0	0
	**MIXED**	0	0	0	0	5 (1)	0	0	0	0	0
**MDH**	**WILD**			100 (28)	100 (22)	100 (54)			100 (28)	100 (21)	100 (58)
	**MUTANT**			0	0	0			0	0	0
	**MIXED**			0	0	0			0	0	0
