# Supplementary material for: Trends in drug resistance codons in Plasmodium falciparum dihydrofolate reductase and dihydropteroate synthase genes in Kenyan parasites from 2008 to 2012
Source: Malar J. 2014 Jul 2;13:250. doi: 10.1186/1475-2875-13-250 (PMC4094641; doi:10.1186/1475-2875-13-250)
Supplement: Additional file 2 — Prevalence of combined mutation in the Pfdhfr and Pfdhps genes. Description: The data presented represent prevalence of haplotypes in the Pfdhfr and Pfdhps genes. [file 1475-2875-13-250-S2.pdf]

Additional file 2: Prevalence of combined mutation in the Pfdhfr and Pfdhps genes

DHFR (MUTANT CODONS UNDERLINED)						DHPS (MUTANT CODONS UNDERLINED)					
ALL SITES	ALL SITES		PER SITE			ALL SITES	ALL SITES		PER SITE		
	COUNTS	%		COUNTS	%		COUNTS	%		COUNTS	%
51/59/108/164 SINGLE	1	0.26	KDH	0	0.00	436/437/540/581 SINGLE	1	0.26	KDH	0	0.00
			KSI	0	0.00				KSI	0	0.00
			KCH	1	1.49				KCH	0	0.00
			MDH	0	0.00				MDH	1	1.23
51/59/108/164 DOUBLE	1	0.26	KDH	0	0.00	436/437/540/581 SINGLE	3	0.78	KDH	0	0.00
			KSI	0	0.00				KSI	0	0.00
			KCH	0	0.00				KCH	0	0.00
			MDH	1	1.23				MDH	3	3.70
51/59/108/164 QUADRUPLE	1	0.26	KDH	1	0.57	436/437/540/581 DOUBLE	3	0.78	KDH	3	1.71
			KSI	0	0.00				KSI	0	0.00
			KCH	0	0.00				KCH	0	0.00
			MDH	0	0.00				MDH	0	0.00
51/59/108/164 TRIPLE	2	0.52	KDH	1	0.57	436/437/540/581 TRIPLE	4	1.03	KDH	4	2.29
			KSI	1	1.56				KSI	0	0.00
			KCH	0	0.00				KCH	0	0.00
			MDH	0	0.00				MDH	0	0.00
51/59/108/164 SINGLE	3	0.78	KDH	0	0.00	436/437/540/581 TRIPLE	6	1.55	KDH	5	2.86
			KSI	0	0.00				KSI	1	1.56
			KCH	0	0.00				KCH	0	0.00
			MDH	3	3.70				MDH	0	0.00
51/59/108/164 DOUBLE	6	1.55	KDH	3	1.71	436/437/540/581 SINGLE	20	5.17	KDH	9	5.14
			KSI	0	0.00				KSI	8	12.50
			KCH	0	0.00				KCH	0	0.00
			MDH	3	3.70				MDH	3	3.70
51/59/108/164 DOUBLE	34	8.79	KDH	5	2.86	437/540/581 DOUBLE	340	87.86	KDH	152	86.86
			KSI	7	10.94				KSI	55	85.94
			KCH	5	7.46				KCH	65	97.01
			MDH	17	20.99				MDH	68	83.95
51/59/108/164 TRIPLE	335	86.56	KDH	164	93.71						
			KSI	56	87.50						
			KCH	60	89.55						
			MDH	55	67.90						
