# Supplementary material for: Trends in drug resistance codons in Plasmodium falciparum dihydrofolate reductase and dihydropteroate synthase genes in Kenyan parasites from 2008 to 2012
Source: Malar J. 2014 Jul 2;13:250. doi: 10.1186/1475-2875-13-250 (PMC4094641; doi:10.1186/1475-2875-13-250)
Supplement: Additional file 3 — Prevalence of Pfdhfr/Pfdhps combined mutations per site (SITE MUTATION/SITE TOTAL*100). Description: The data represents the prevalence of mutation occurring in both Pfdhfr and Pfdhps genes simultaneously. [file 1475-2875-13-250-S3.pdf]

Additional File 3: Prevalence of Pfdhfr/Pfdhps combined mutations per site (SITE MUTATION/SITE TOTAL*100)

COMBINED DHFR-DHPS MUTATIONS (MUTANT CODONS UNDERLINED)					
**ALL SITES**			**PER SITE**		
	**COUNTS**	**%**		**COUNTS**	**%**
51/59/108/164/436/437/540/581 QUADRUPLE	1	0.26	KDH	0	0.0
			KSI	0	0.0
			KCH	0	0.0
			MDH	1	1.2
51/59/108/164/436/437/540/581 SEXTUPLE	1	0.26	KDH	1	0.6
			KSI	0	0.0
			KCH	0	0.0
			MDH	0	0.0
51/59/108/164/436/437/540/581 QUINTUPLE	2	0.52	KDH	1	0.6
			KSI	1	1.6
			KCH	0	0.0
			MDH	0	0.0
51/59/108/164/436/437/540/581 QUADRUPLE	3	0.78	KDH	0	0.0
			KSI	0	0.0
			KCH	0	0.0
			MDH	3	3.7
51/59/108/164/436/437/540/581 QUINTUPLE	3	0.78	KDH	3	1.7
			KSI	0	0.0
			KCH	0	0.0
			MDH	0	0.0
51/59/108/164/436/437/540/581 QUINTUPLE	6	1.55	KDH	5	2.9
			KSI	1	1.6
			KCH	0	0.0
			MDH	0	0.0
51/59/108/164/436/437/540/581 QUADRUPLE	19	4.91	KDH	9	5.1
			KSI	8	12.5
			KCH	0	0.0
			MDH	2	2.5
51/59/108/164/436/437/540/581 QUINTUPLE	293	75.71	KDH	141	80.6
			KSI	48	75.0
			KCH	60	89.6
			MDH	44	54.3
