# Supplementary material for: Trends in drug resistance codons in Plasmodium falciparum dihydrofolate reductase and dihydropteroate synthase genes in Kenyan parasites from 2008 to 2012
Source: Malar J. 2014 Jul 2;13:250. doi: 10.1186/1475-2875-13-250 (PMC4094641; doi:10.1186/1475-2875-13-250)
Supplement: Additional file 4 — Percentage temporal prevalence of genotypes. Description: The data shows average genotype prevalence of all the study sites over the period under investigation. The genotypes analysed for Pfdhfr were N51I, C59R, S108N and I164L while for Pfdhps were S436A, A437G, K540E and A581G in that order. Amino acid codes before the loci position indicate the wildtype while the ones indicated after the loci position are the mutations. In the table, the genotypes without neither bold nor underlined amino acid codes are the wild type while the underlined and bold are the mutant alleles. [file 1475-2875-13-250-S4.pdf]

Additional file 4: Percentage temporal prevalence of genotypes

		**2008**	**2009**	**2010**	**2011**	**2012**
Pfdhfr	NCSI	0.0	1.1	1.3	2.9	0.0
	N**R**SI	0.0	0.0	1.3	0.0	0.0
	**IR**SI	0.0	0.0	0.0	1.4	0.0
	**IRNL**	0.0	0.0	0.0	0.0	1.2
	**I**C**NL**	3.0	0.0	0.0	0.0	0.0
	NC**N**I	0.0	0.0	1.3	0.0	2.4
	N**RN**I	3.0	0.0	2.6	1.4	1.2
	**I**C**N**I	1.5	6.6	1.3	10.1	22.9
	**IRN**I	92.5	92.3	92.2	84.1	72.3
Pfdhps	SAKA	0.0	1.1	9.1	2.9	0.0
	**A**AKA	0.0	0.0	0.0	1.4	0.0
	SA**E**A	0.0	0.0	0.0	1.4	2.4
	S**G**K**G**	1.5	1.1	1.3	0.0	0.0
	**AGE**A	1.5	3.3	0.0	0.0	0.0
	S**GEG**	0.0	0.0	0.0	0.0	7.2
	S**G**KA	6.0	6.6	6.5	4.3	2.4
	S**GE**A	91.0	87.9	83.1	89.9	88.0
